# Short Account of Addenbrooke's Hospital, Cambridge

**Published:** 1805-10-01

**Authors:** 


### Short Account of Addenbrooke's Hospital, Cambridge.

In the year 1719, John Addenbrooke, M.D. formerly of Catharine Hall in this University, left about £.4000 to erect and maintain a small physical hospital. Land was accordingly purchased in 1728, and the building erected in 1740, but the money being insufficient for the support of it, an Act of Parliament was obtained in the year 1766, for making it a General Hospital, and in October of the same year it was first opened for patients. It has been supported by legacies, benefactions, and annual voluntary contributions, an annual statement of which, and the expences, are given to the public. It is situated at the south entrance of the town from London, and is a modern and commodious building of brick, standing in the midst of a garden, with a stream of water in the front of it. The kitchen, kitchen, cellar, cold bath, hot bath, and other offices, are under ground; on the first floor are two wards, (one for men, the other for women) the apothecary's shop, and matron's room; on the second floor are two more wards, and a handsome committee room; on the attic story are bed rooms for the apothecary, matron, and servants, and a small ward for patients; each ward is capable of holding twelve patients and a nurse, and has every accommodation for comfort and conveniency, and the whole house exhibits a specimen of neatness and good order, scarcely to be equalled, certainly not excelled.

The matron and apothecary lodge and board in the house, having each a salary, with servants and nurses under their direction. The President is the present Lord Lieutenant of Ireland, and the Governors consist of the higher officers of the University, county and town of Cambridge, and such persons as annually subscribe two guineas and upwards.

The present Officers of the Charity are,

John Newling, Esq. Treasurer, elected 1771.

Physicians and Surgeons who attend *gratis.*

Sir Isaac Pennington, Knt. M.D. Regius Professor of Physic, elected 1773.

Busick Harwood, M.D. Professor of Anatomy, elected 1786.

Robert Stockdale, A.M. L.M. elected 1791.

Thomas Ingle, M.D. elected 1791.

Thomas Thackeray, Esq. consulting Surgeon, elected 1766.

Thomas Bond, Esq. elected 1776 }
Thomas Verney Okes, Esq. elected 1776 } Surgeons.
Frederic Thackery, Esq. elected 1796 }

Mr. Robert Gee, Secretary, with salary, elected 1775.

Mr. Joseph Gray, Apothecary, ditto, elected 1784.

Mrs. Charlotte Weybrew, Matron, ditto, elected 1802.

List of Patients for the last seven Years.

| | In patients admitted. | Out Patients admitted. | Patients cured. |
|---|---|---|---|
| 1798 | 336 | 402 | 446 |
| 1799 | 302 | 363 | 421 |
| 1800 | 308 | 395 | 446 |
| 1801 | 323 | 419 | 478 |
| 1802 | 309 | 412 | 467 |
| 1803 | 294 | 339 | 468 |
| 1804 | 303 | 349 | 434 |

The

The total number of the patients cured since the opening of the Hospital in the year 1766, has been 16087; of the remainder, some have been relieved, in-patients made out-patients, out-patients made in-patients, some discharged at their own request, or for irregularity, &c. &c.

